# Circadian Reprogramming of Protein Homeostasis and Glycolipid Metabolism in Diabetic Nephropathy

**DOI:** 10.1096/fba.2025-00305

**Published:** 2026-02-11

**Authors:** Xiao‐Qian Li, Lei Cheng, Tian‐Fen Chen, Yi‐Nuo Ma, Lu‐Yao Wang, Xiao‐Hui Li, Ting‐Yu Fu, Jing Xiao, Zhan‐Zheng Zhao

**Affiliations:** ^1^ Nephrology Hospital, The First Affiliated Hospital of Zhengzhou University Zhengzhou University Zhengzhou Henan China; ^2^ Zhengzhou University Zhengzhou Henan China; ^3^ Laboratory Animal Platform of Academy of Medical Sciences Zhengzhou University Zhengzhou Henan China

**Keywords:** circadian, diabetic nephropathy, fatty acid metabolism, glycolipid metabolism, protein homeostasis

## Abstract

Dysfunction of the circadian clock has been implicated in the pathogenesis of various diseases, including metabolic disorders, inflammatory conditions, and cancer. While the significance of circadian rhythm in diabetic nephropathy is gaining attention, the specific alterations in circadian profiles in diabetic nephropathy remain unexplored. In the present study, we performed RNA sequencing on renal cortex samples collected every 4 h across the day from both control and diabetic mice. The rhythmicity of genes was identified using the JTK_CYCLE algorithm for each group. Genes that lost, acquired, or sustained rhythmicity in diabetic mice were denoted the circadian dysregulation gene set. Subsequent bioinformatic analyses focused on this gene set to investigate the circadian reprogramming in diabetic nephropathy. We observed significant circadian disruption in the kidney of diabetic mice, marked by both the gain and loss of rhythmicity, along with alterations in the phase and relative amplitude of genes that retained rhythmic expressions. Circadian disturbances, such as phase shifts and alterations in relative amplitude or mesor, were also noted in core clock genes. Furthermore, genes that lost rhythmicity in diabetic nephropathy were predominantly associated with protein homeostasis and glycolipid metabolism, whereas those that gained rhythmicity were mainly linked to gene regulation, fatty acid metabolism, and protein transport. The genes in the circadian dysregulation gene set that exhibit differential expression at least at one Zeitgeber time were most prominently enriched in the lipid metabolic process. WGCNA and correlation analysis revealed co‐expression networks involving core clock genes and PPAR signaling pathway with renal triglyceride levels. Our study reveals substantial circadian disruption in diabetic nephropathy, with significant impacts on protein homeostasis and glycolipid metabolism. Furthermore, our findings highlight the potential influence of circadian system dysregulation on the disorder of fatty acid metabolism in diabetic nephropathy.

## Graphical abstract

Using time‐series transcriptomic profiling of diabetic mouse kidneys, our study reveals substantial circadian disruption in diabetic nephropathy, with significant impacts on protein homeostasis and glycolipid metabolism. Furthermore, our findings highlight the potential influence of circadian system dysregulation on the disorder of fatty acid metabolism in diabetic nephropathy.

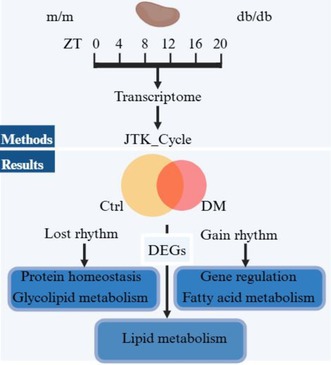

## 1 Introduction

Diabetic nephropathy, a primary microvascular complication of diabetes mellitus, stands as the predominant cause of end‐stage renal disease globally [1, 2]. Given the limited treatment strategies available, further exploration is required to expand our inadequate comprehension of the pathogenesis underlying diabetic nephropathy.

The internal timekeeping system, referred to as the circadian clock, is a rhythmic and evolutionarily preserved mechanism that could synchronize physiological and behavioral processes in alignment with external cues like daylight, ambient temperature, and dietary habits. The autonomous circadian rhythm is orchestrated by two principal transcription factors: the clock circadian regulator (CLOCK) and the basic helix–loop–helix ARNT‐like 1 (BMAL1). These two proteins combine to form a complex that triggers the expression of core clock genes, including the period1/2/3 (PER1/2/3), cryptochrome1/2 (CRY1/2), nuclear receptor subfamily 1, group D, members 1 and 2 (NR1D1/2), and RAR‐related orphan receptors α/β/γ (RORα/β/γ), etc. These genes jointly constitute the transcription‐translation feedback network, which in turn modulates the transcription of downstream circadian‐controlled genes.

Acting as the conductor of the body, the circadian clock is intricately interconnected with a multitude of biological functions. Disturbances of the circadian system could result in a spectrum of diseases, including metabolic disorders [3], inflammatory diseases [4], and cancer [5]. The circadian clock lies in the suprachiasmatic nucleus of the central nervous system as well as the peripheral organs like the kidney. Zhang et al. [6] detected that the proportion of rhythmic genes in the kidney was about 13%, only surpassed by the liver (about 16%). Indeed, the key renal physiological processes such as electrolyte excretion, blood pressure control, and hormone production are circadian controlled [7], highlighting the substantial influence of circadian rhythm on kidney function.

The potential influence of the circadian clock in diabetic nephropathy is increasingly recognized through a range of clinical investigations. Epidemiological research has demonstrated that sleep circadian disturbance is closely related to elevated risk of metabolic syndrome, including obesity and type 2 diabetes [8]. Besides, investigators further established a link between circadian disturbance of blood pressure and sleep behavior with the progression of chronic kidney disease and diabetic nephropathy [9, 10, 11, 12]. In recent years, several researchers have begun to take an interest in the significance of the intrarenal circadian clock in diabetic nephropathy. Research indicates that dysfunction of the circadian system might lower the threshold of kidney disease or worsen its progression. For instance, in normoglycemic mice, nephron‐specific deletion of *Bmal1* resulted in substantial alterations in renal metabolic pathways and a significant decrease in the nicotinamide adenine dinucleotide oxidized‐to‐reduced ratio, which reflects the mitochondrial function [13]. Ansermet and colleagues found that specific knockdown of *Bmal1* in renal tubular cells could lead to elevated blood glucose in diabetic mice by enhancing renal gluconeogenesis [14]. Furthermore, a recently published study conducted by Wang et al. [15] validated that podocyte autophagy is regulated by *Clock*, and deletion of *Clock* in podocytes led to increased podocyte damage and elevated proteinuria in diabetic mice, shedding light on the potential pathogenic significance of the *clock* in diabetic nephropathy.

While studies have begun to explore the dysfunction of core clock genes like *Bmal1* and *Clock* in diabetic nephropathy, the extent to which the circadian system is influenced in diabetic kidney remains unclear. Furthermore, the consequences of circadian disturbance on downstream rhythmic biological functions in the context of diabetic nephropathy are yet to be fully elucidated. Here we performed the RNA sequencing on the renal cortex collected every 4 h across the day from both control and diabetic mice, followed by comprehensive bioinformatic analyses.

## 2 Materials and Methods

### 2.1 Animal Experiments

Ten‐week‐old male C57BLKS/J background m/m and db/db mice were supplied by GemPharmatech Co. Ltd. (Nanjing, China). Mice were accommodated in the Laboratory Animal Center of Henan Province with a 12‐h light/12‐h dark cycle (light on at 8:00 AM was specified as zeitgeber time (ZT) 0 and light off at 8:00 PM was defined as ZT12). They had unrestricted access to food and water. Blood glucose was measured on tail‐vein blood samples using a glucose analyzer (Accu‐check Performa; Roche, Basel, Switzerland). Mice were euthanized at 21 weeks of age by cervical dislocation starting at ZT0 with 4‐h intervals for 24 h. Kidney tissues were harvested every 4 h, rapidly frozen in liquid nitrogen, and then stored at −80°C. All manipulations during the night were performed under a 15‐W red light. This study was approved by the Animal Ethics Committee of the First Affiliated Hospital of Zhengzhou University (approval No. ZZU‐LAC20210604[09]).

### 2.2 RNA Isolation and Library Preparation

At ZT8, the control group mice had three biological replicates, while all other ZTs for both the control and diabetic groups included four replicates. The RNA isolation and library preparation were handled by OE Biotech Co. Ltd. (Shanghai, China). The mirVana miRNA Isolation Kit (Catalog No. AM1561; Thermo Fisher Scientific, Massachusetts, USA) was utilized to extract total RNA, following the protocol provided by the manufacturer. The integrity of the RNA was assessed with the Agilent 2100 Bioanalyzer (Agilent Technologies, Santa Clara, CA, USA). Only samples with RNA integrity number ≥ 7 were advanced for further analyses. RNA concentrations were determined using the NanoDrop 2000 spectrophotometer (Thermo Fisher Scientific, MA, USA). Library construction was performed with the TruSeq Stranded mRNA LTSample Prep Kit (Illumina, San Diego, CA, USA).

### 2.3 RNA Sequencing

OE Biotech Co. Ltd. (Shanghai, China) conducted the transcriptome profiling and analyses. In brief, the libraries were processed and sequenced with the Illumina sequencing system. The raw sequencing data were processed using Trimmomatic to remove low‐quality reads and those containing ploy‐N sequences, resulting in clean reads. The fragments per kilobase million (FPKM) value of each gene was calculated using cufflinks, and the read count of each gene was obtained by htseq‐count. The raw and normalized transcriptome data in this work were submitted to NCBI, which can be accessed at https://www.ncbi.nlm.nih.gov/geo/query/acc.cgi?acc=GSE283571.

### 2.4 Circadian Rhythm Analysis

The rhythmicity of transcriptome genes in both control and diabetic mice was analyzed using JTK_CYCLE [16] (MetaCycle package in R), with the Benjamini‐Hochberg correction applied to adjust for multiple comparisons and control the false discovery rate. A threshold of *p*‐value < 0.01 was established as the criterion for determining rhythmicity. The relative amplitude was calculated as the ratio between amplitude and baseline of the cosine fit. The phase was defined as the time of the peaking rhythm. Both relative amplitude and phase were analyzed by JTK_CYCLE. For comparative analysis of circadian parameters of core clock genes between the two groups, Cosinor analysis was employed utilizing an online tool (https://cosinor.online/app/cosinor.php).

### 2.5 Bioinformatics Analyses

Genes were categorized into four distinct datasets according to the changes in their rhythmic patterns between control and diabetic mice: (1) genes that exhibited rhythmicity in both groups, (2) genes that were rhythmic in control but lost rhythmicity in diabetic mice, (3) genes arrhythmic in control mice but gained rhythmicity in diabetic mice, and (4) genes that were arrhythmic under both conditions. Single‐cell RNA‐seq dataset of diabetic nephropathy (GSE131882) was downloaded from GEO. Core cell populations were annotated using the SingleR R package. The expression scores for distinct rhythmic gene sets were computed using the AddModuleScore in the Seurat R package. We utilized the fuzzy c‐means method within the Mfuzz R package to categorize genes into clusters based on their circadian expression profiles. The Z‐score‐transformed RPKM value for each gene at each time point was used as the input. ClueGO analysis was performed utilizing the Cytoscape ClueGO plug‐in to visualize non‐redundant biological terms linked to large gene clusters, organized into a functional network. Gene Ontology (GO) and Kyoto Encyclopedia of Genes and Genomes (KEGG) pathway analyses, along with Weighted Gene Co‐expression Network Analysis (WGCNA), correlation matrix plot, heat map, and different types of bubble plots were conducted using the online tools from OE Cloud Platform (https://cloud.oebiotech.cn/#/home) and Wei Sheng Xin platform (https://www.bioinformatics.com.cn/). Enrichment terms with *p*_adj_ value < 0.05 were considered statistically significant.

### 2.6 Periodic Acid‐Schiff (PAS) Staining

The paraffin embedding and sectioning were carried out by the Laboratory Animal Center of Henan Province. PAS staining was performed following the manufacturer's instructions (BA4114B, BaSO, Zhuhai, China) and evaluated as previously described [17]. Representative histological morphologies of mice were captured under 200× and 400× magnification.

### 2.7 Biochemical Assays

Mice were housed in a 24‐h metabolic chamber for urine collection, and urinary albumin concentrations were determined by a mouse albumin ELISA kit (Bethyl Laboratories, Montgomery, TX, USA). Additionally, urinary creatinine levels were quantified by a Creatinine Assay Kit (DICT‐500; BioAssay Systems, CA, USA). The ratio of urinary albumin to creatinine was calculated to correct the difference in urine volume and expressed in units of micrograms per milligram. Plasma levels of triglycerides and total cholesterol, as well as renal triglyceride levels, were measured using commercial assay kits (A110‐1‐1 and A111‐1‐1, respectively) from Nanjing Jiancheng Bioengineering Institute (Nanjing, China). Renal triglyceride was extracted by an ultrasonic homogenizer and corrected by the renal protein level.

### 2.8 Statistical Analyses

Data are displayed as mean ± SEM. Student's *t*‐test was used to compare independent continuous variables between two groups. ZT‐dependent circadian gene expression differences between control and diabetic mice were analyzed by two‐way ANOVA followed by Bonferroni multiple comparison correction using GraphPad Prism software version 7 (GraphPad Software Inc., CA, USA). *p* < 0.05 was considered statistically significant.

## 3 Results

### 3.1 The Renal Circadian Profile Is Reprogrammed in Diabetic Nephropathy

**FIGURE 1:**
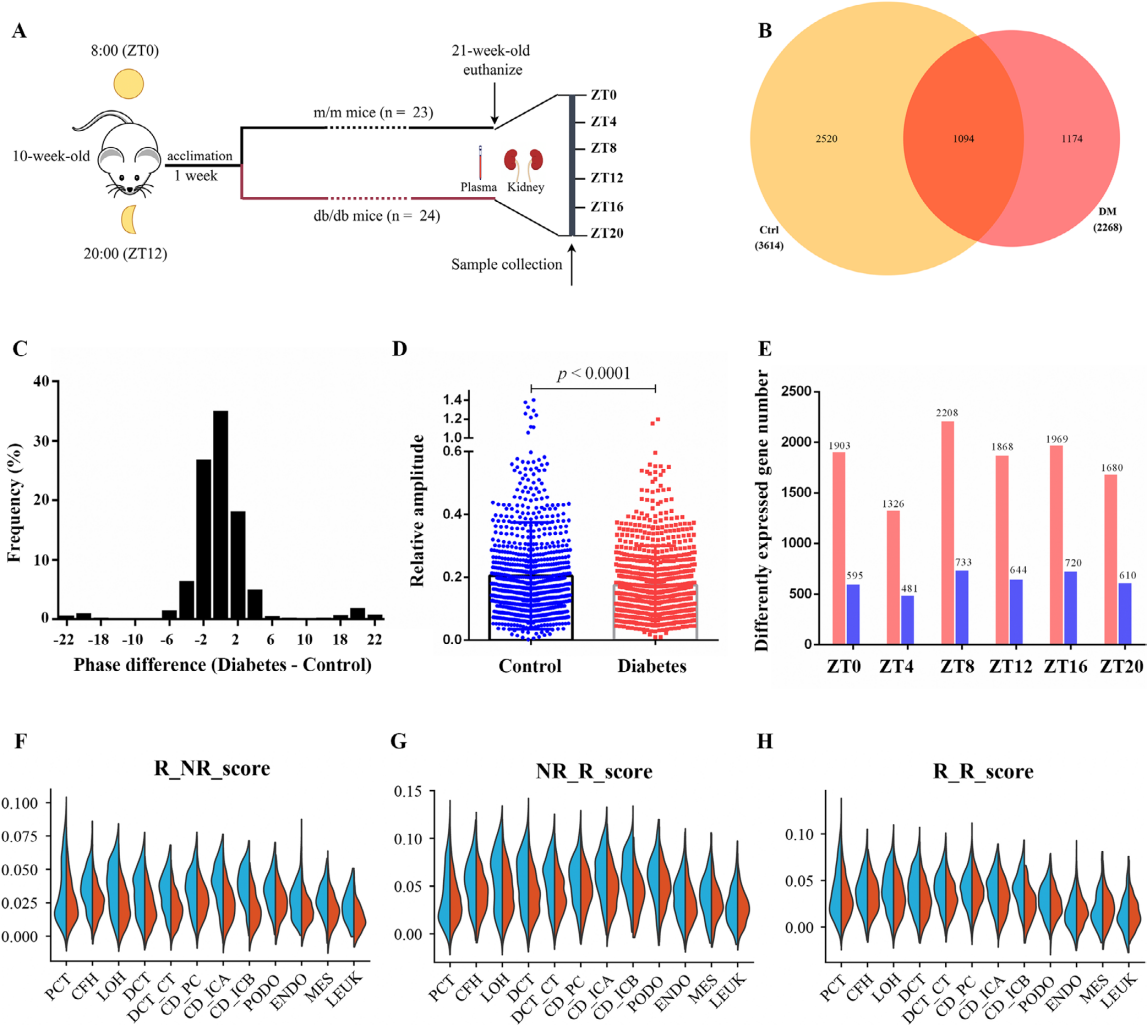
The renal circadian transcription profile alteration in diabetic mice. Overview of the animal experimental design. (B) Venn diagram displaying the number of rhythmic genes identified by JTK_CYCLE in the kidneys of control and diabetic mice. (C) Histogram representing the distribution of phase difference (phase of diabetic minus the phase of control) of the 1094 genes remained rhythmic in both conditions. (D) Relative amplitudes of the 1094 genes remained rhythmic in control and diabetic mice. Data are expressed as mean ± SEM. (E) Differentially expressed genes between diabetes and control across different ZTs. The red bars indicate the upregulated gene number, while the blue bars indicate the downregulated gene number. Violin plots display the expression scores of genes losing (F), gaining (G), or retaining (H) circadian rhythmicity across kidney‐cell clusters in control (blue) and diabetic (red) mice. ZT, zeitgeber time.

The overall design scheme is displayed in Figure 1A. The db/db mice euthanized at 21 weeks of age developed typical characteristics of type 2 diabetes, including hyperglycemia, hypercholesterolemia, and hypertriglyceridemia (Figure S1A–C). Besides, the levels of urinary albumin creatinine ratio of db/db mice were significantly higher than those of m/m mice (Figure S1D). PAS staining was performed to verify the diabetic renal damage such as glomerular hypertrophy, mesangial matrix expansion, and tubulointerstitial injury (Figure S1E), validating the progression of diabetic nephropathy in db/db mice.

A total of 3614 genes displayed circadian rhythmicity in the kidney of control mice (16% of the total mRNA), and a total of 2268 rhythmic genes were recognized in diabetic mice (10% of the total mRNA). 30% of the oscillating genes in control mice (1094 out of 3614) maintained rhythmicity, while 70% of the oscillating genes in control mice (2520 out of 3614) lost rhythmicity in diabetic mice. In addition, 1174 genes that were not rhythmic in control mice gained rhythmicity in diabetic kidney (Figure 1B). Regarding the 1094 genes oscillating in both conditions, 65% of genes showed phase advance or phase delay (phase of diabetes—phase of control) (Figure 1C). Besides, the relative amplitudes of the 1094 genes in diabetic mice declined in diabetic mice compared with the control (0.175 ± 0.003 vs. 0.205 ± 0.005, *p* < 0.0001, Figure 1D). In addition, we found the total differential genes between diabetes and control varied across different ZTs. Notably, the disparity was most pronounced at ZT8 (Figure 1E). The above results indicate that the renal circadian profile is reprogrammed in diabetic nephropathy. To identify the renal cellular source of the diabetes‐reprogrammed circadian signature, we interrogated the publicly available single‐cell RNA‐seq dataset of diabetic nephropathy (GSE131882). Expression scores for gene sets that lose, gain, or retain circadian rhythmicity were calculated in each kidney‐cell cluster. We found the tubular cells exhibited higher expression scores than the glomerular cells, indicating that the reprogrammed circadian profile originates predominantly from tubular cells under diabetic conditions (Figure 1F–H).

### 3.2 The Circadian Patterns of Renal Core Clock Genes Are Disturbed in Diabetic Nephropathy

**FIGURE 2:**
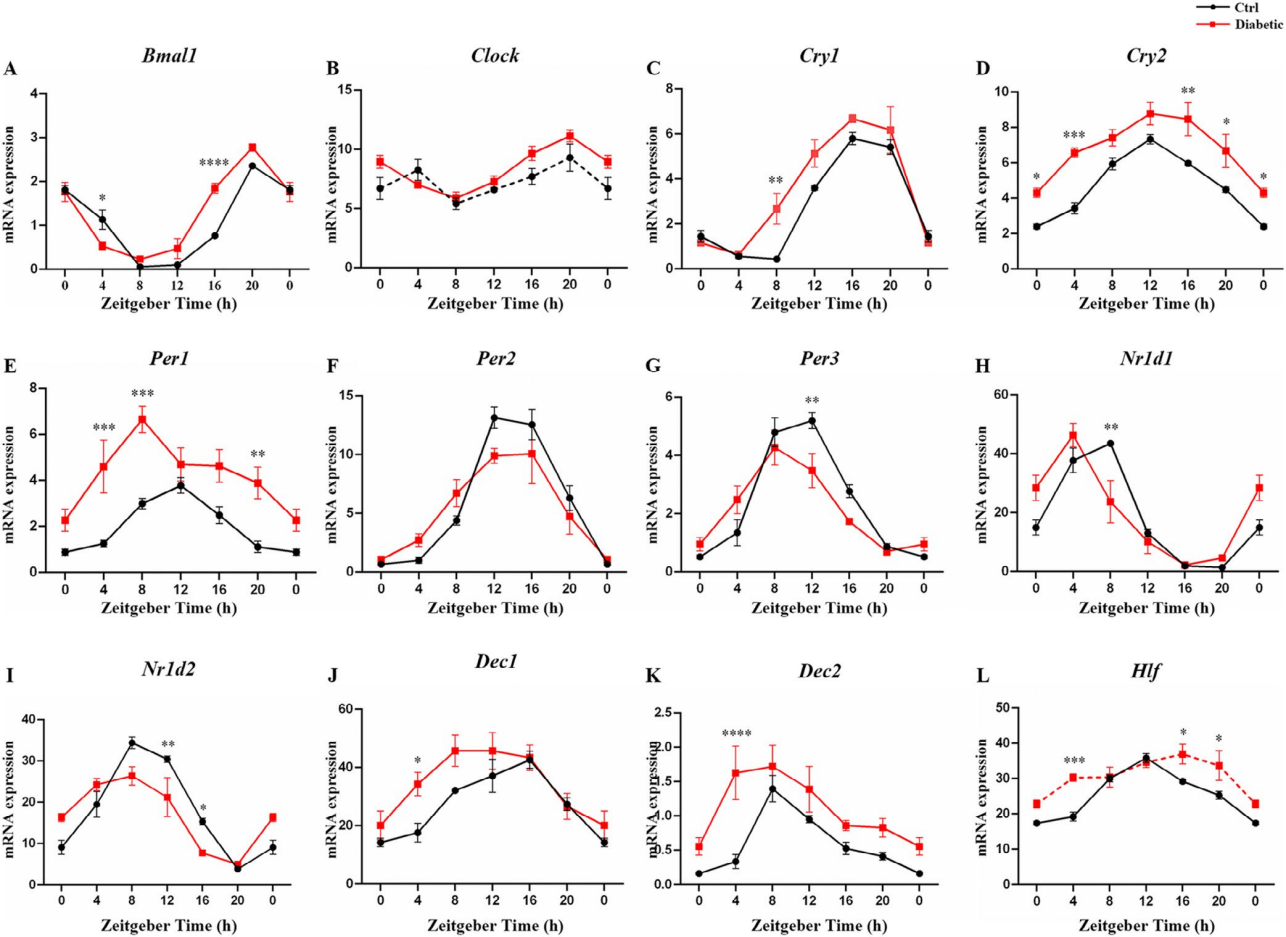
The circadian patterns of renal core clock genes in diabetic mice. The renal circadian expressions of core clock genes including (A) *Bmal1*, (B) *Clock*, (C) *Cry1*, (D) *Cry2*, (E) *Per1*, (F) *Per2*, (G) *Per3*, (H) *Nr1d1*, (I) *Nr1d2*, (J) *Dec1*, (K) *Dec2* and (L) *Hlf* in control (black line) and diabetic (red line) mice during 24 h. The solid line denotes rhythmic oscillation, while the dotted line represents arrhythmic. Data are mean ± SEM. Two‐way ANOVA with Bonferroni multiple comparison correction was performed, and *p*‐value < 0.05 was considered significant. *p < 0.05; **p < 0.01; ***p < 0.001; ****p < 0.0001.

To examine whether the differential renal circadian expression profile between control and diabetic mice was attributed to the intra‐renal circadian clock, we next analyzed the core clock regulatory network. As shown in Figure 2, *Bmal1*, *Cry1*, *Cry2*, *Per1*, *Per2*, *Per3*, *Nr1d1*, *Nr1d2*, *Dec1*, and *Dec2* showed strong oscillation both in diabetic and control mice, while *Clock* gained and *Hlf* lost rhythmicity in diabetes. Cosinor analysis revealed phase advancement of *Bmal1*, *Per3*, *Nr1d1*, *Nr1d2*, and *Dec1* in diabetic mice. Additionally, the mesor of *Bmal1*, *Cry1*, *Cry2*, *Per1*, *Dec1*, and *Dec2* was significantly elevated in diabetic mice, whereas the amplitude of *Nr1d2* was markedly reduced (Table S1). Moreover, several clock genes (*Bmal1*, *Cry1*, *Cry2*, *Per1*, *Per3*, *Nr1d1*, *Nr1d2*, *Dec1*, *Dec2*, and *Hlf*) exhibited differential expressions at one or more ZTs.

### 3.3 The Circadian of Protein Homeostasis and Glycolipid Metabolism Is Disrupted in Diabetic Nephropathy

**FIGURE 3:**
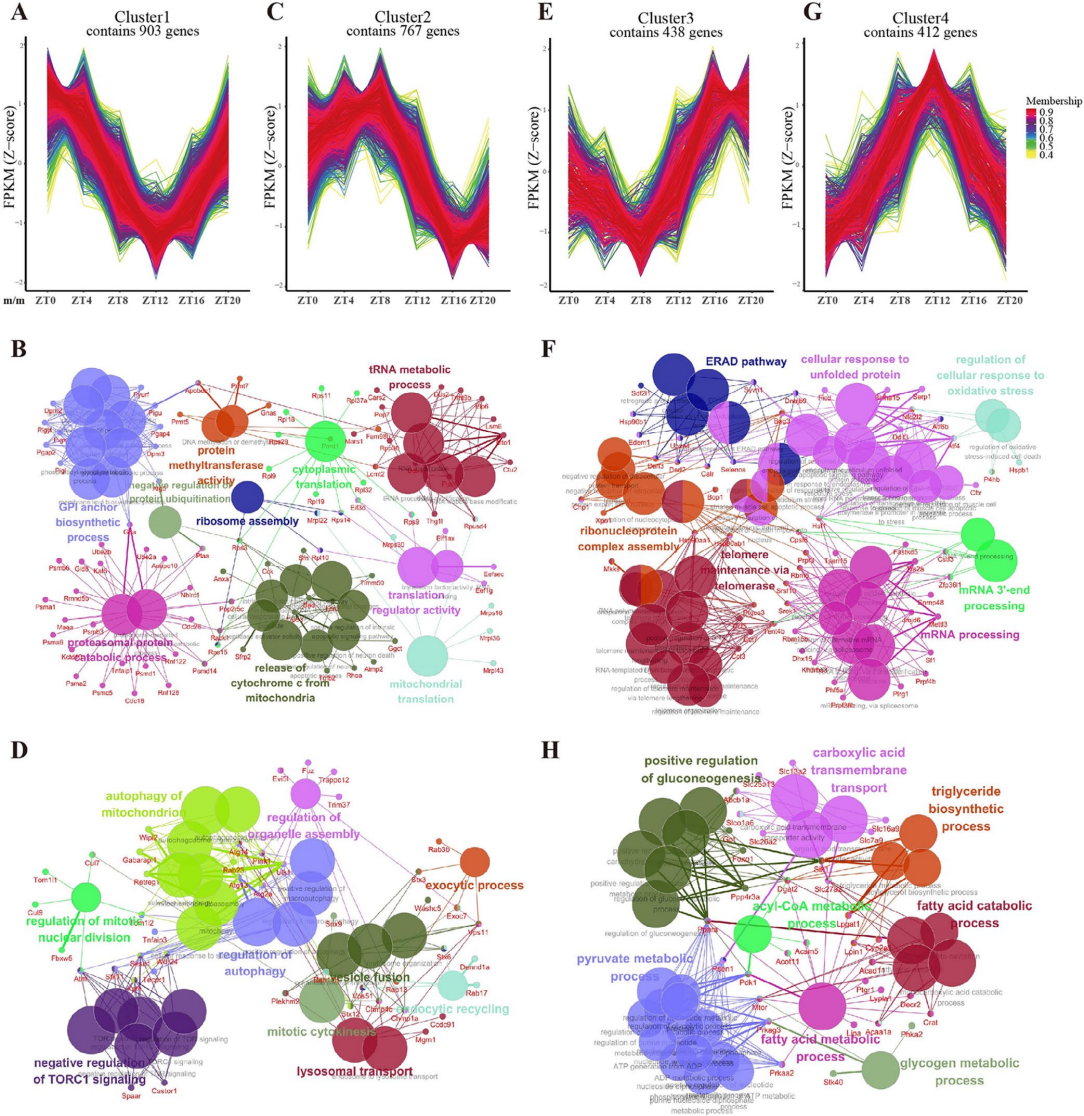
Biological functions of genes with lost rhythmicity in diabetic nephropathy. Cluster 1 plot of the Mfuzz gene clustering analysis for the 2520 genes lost rhythmicity in diabetic nephropathy. Since these genes do not exhibit rhythmicity in the diabetic group, the figure displays only the circadian patterns of genes in control mice. This situation is consistent across the other clusters as well. *x* axis, different ZTs as labeled; *y* axis, the Z‐score‐transformed FPKM values. (B) ClueGO analysis visualizes the connection network among biological functions of Cluster 1 genes. Biological processes that are functionally related are color‐coded identically. The size of each node corresponds to the significance of term enrichment, with only the most significant term's label being displayed per group. Related terms may share similar associated genes. (C) Mfuzz gene clustering analysis plot and (D) ClueGO analysis of Cluster 2 genes lost rhythmicity in diabetic nephropathy. (E) Mfuzz gene clustering analysis plot and (F) ClueGO analysis of Cluster 3 genes lost rhythmicity in diabetic nephropathy. (G) Mfuzz gene clustering analysis plot (H) and ClueGO analysis of Cluster 4 genes lost rhythmicity in diabetic nephropathy.

We then focused on genes that lost rhythmicity in diabetic mice. To differentiate distinct oscillatory patterns and gain insights into the function of genes with similar rhythmic profiles, Mfuzz and GO analyses were performed. Then, ClueGO analysis was used to visualize the connections between GO terms. Based on the preliminary analysis, the genes were grouped into four clusters, each representing a unique circadian pattern. Genes in Cluster 1 reached the nadir around ZT12 (Figure 3A). Despite serving diverse functions, these processes are interconnected through roles in protein synthesis, modification, degradation, and cellular signal transduction (Figure 3B). The circadian pattern of Cluster 2 is characterized by a gradual ascent, reaching its zenith in the midst of the resting phase, followed by a descent to its nadir during the latter part of the active phase (Figure 3C). Genes in Cluster 2 were primarily associated with various stages and regulatory mechanisms of autophagy (Figure 3D). Cluster 3 exhibited opposite circadian patterns compared to Cluster 2 (Figure 3E), and the genes in Cluster 3 were mainly engaged in unfolded protein response and the ERAD pathway, which are critical for ensuring proper protein folding and endoplasmic reticulum homeostasis. Besides, these biological processes are coupled with mRNA processing and ribonucleoprotein complex assembly, the essential steps for protein synthesis (Figure 3F). Cluster 4 was predominantly related to glycolipid metabolism, including fatty acid catabolic process, acyl‐CoA metabolic process, positive regulation of gluconeogenesis, and glycogen metabolic process (Figure 3G,H). Collectively, these findings suggest that the circadian regulation of protein homeostasis, encompassing both protein synthesis and degradation, along with glycolipid metabolism, is disrupted in diabetic nephropathy.

### 3.4 Gene Expression Regulation, Lipid Metabolism and Protein Transport Acquire Rhythmicity in Diabetic Nephropathy

**FIGURE 4:**
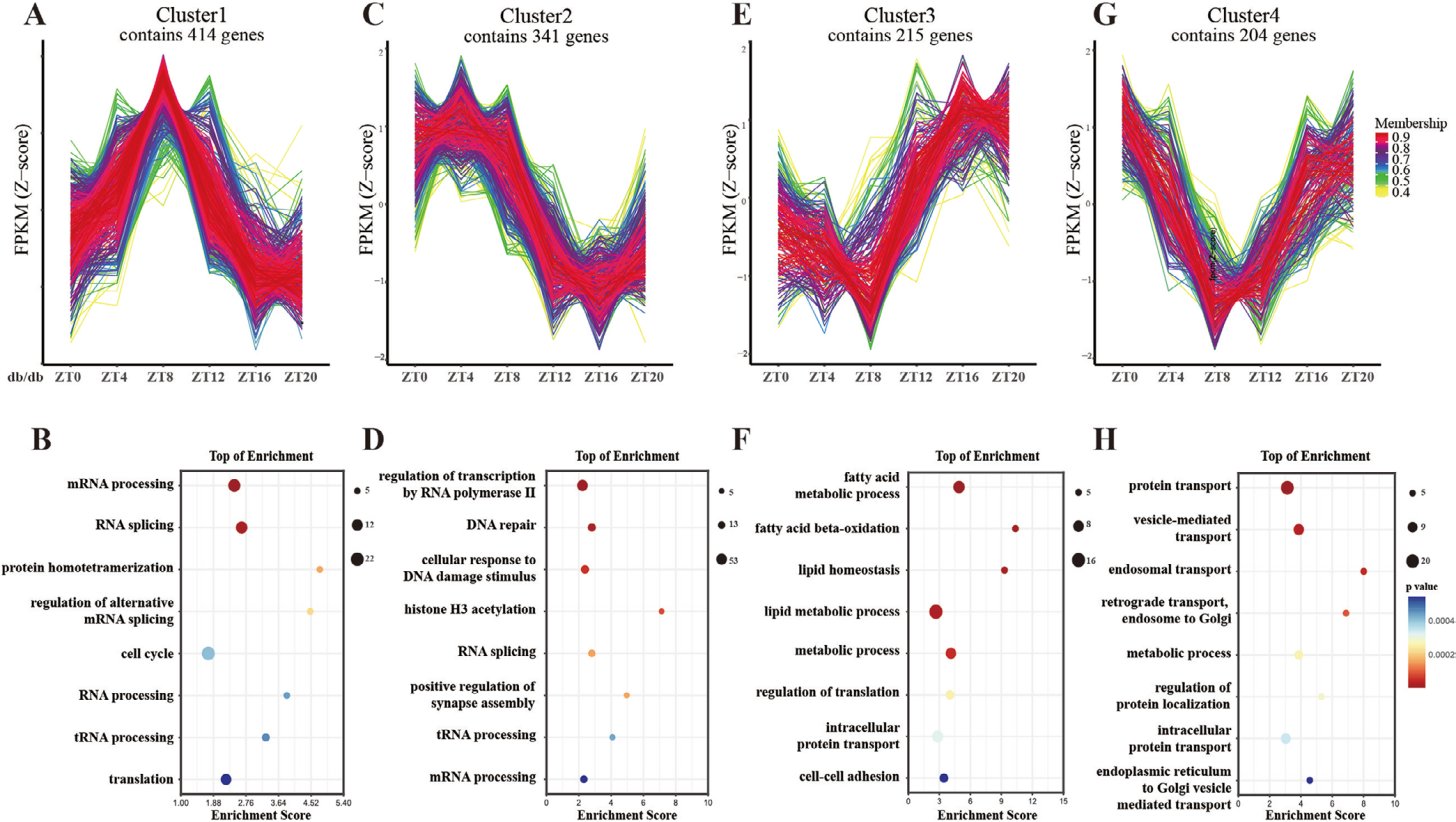
The biological functions of genes acquire rhythmicity in diabetic nephropathy. (A) Cluster 1 plot of the Mfuzz gene clustering analysis for the 1147 genes gain rhythmicity in diabetic nephropathy. Since these genes do not exhibit rhythmicity in the control group, the figure only presents the circadian patterns of genes in diabetic mice. This situation is consistent across the other clusters as well. *x* axis, different ZTs as labeled; *y* axis, the Z‐score‐transformed FPKM values. (B) Top 8 significant biological processes of Cluster 1 genes. (C) Mfuzz gene clustering analysis and (D) top 8 significant enriched biological processes of cluster 2 genes gain rhythmicity in diabetic nephropathy. (E) Mfuzz gene clustering analysis and (F) top 8 significant enriched biological processes of Cluster 3 genes gain rhythmicity in diabetic nephropathy. (G) Mfuzz gene clustering analysis and (H) top 8 significant enriched biological processes of Cluster 4 genes gain rhythmicity in diabetic nephropathy.

Then Mfuzz and GO analyses were conducted on genes that gained rhythmicity in diabetic mice. The Cluster 1 genes encompass key biological processes in gene expression, for instance, mRNA processing, translation, and cell cycle regulation (Figure 4A,B). The Cluster 2 genes were also similarly involved in the regulation of gene expression, encompassing transcription, RNA processing, and translation. Besides, these genes were also associated with DNA repair and cellular damage response (Figure 4C,D). The Cluster 3 genes were mainly linked to the fatty acid metabolic process and lipid homeostasis (Figure 4E,F), while genes in Cluster 4 were mainly associated with intracellular protein transport (Figure 4G,H).

### 3.5 Circadian Dysregulation of Lipid Metabolism Is a Prominent Feature in Diabetic Nephropathy

**FIGURE 5:**
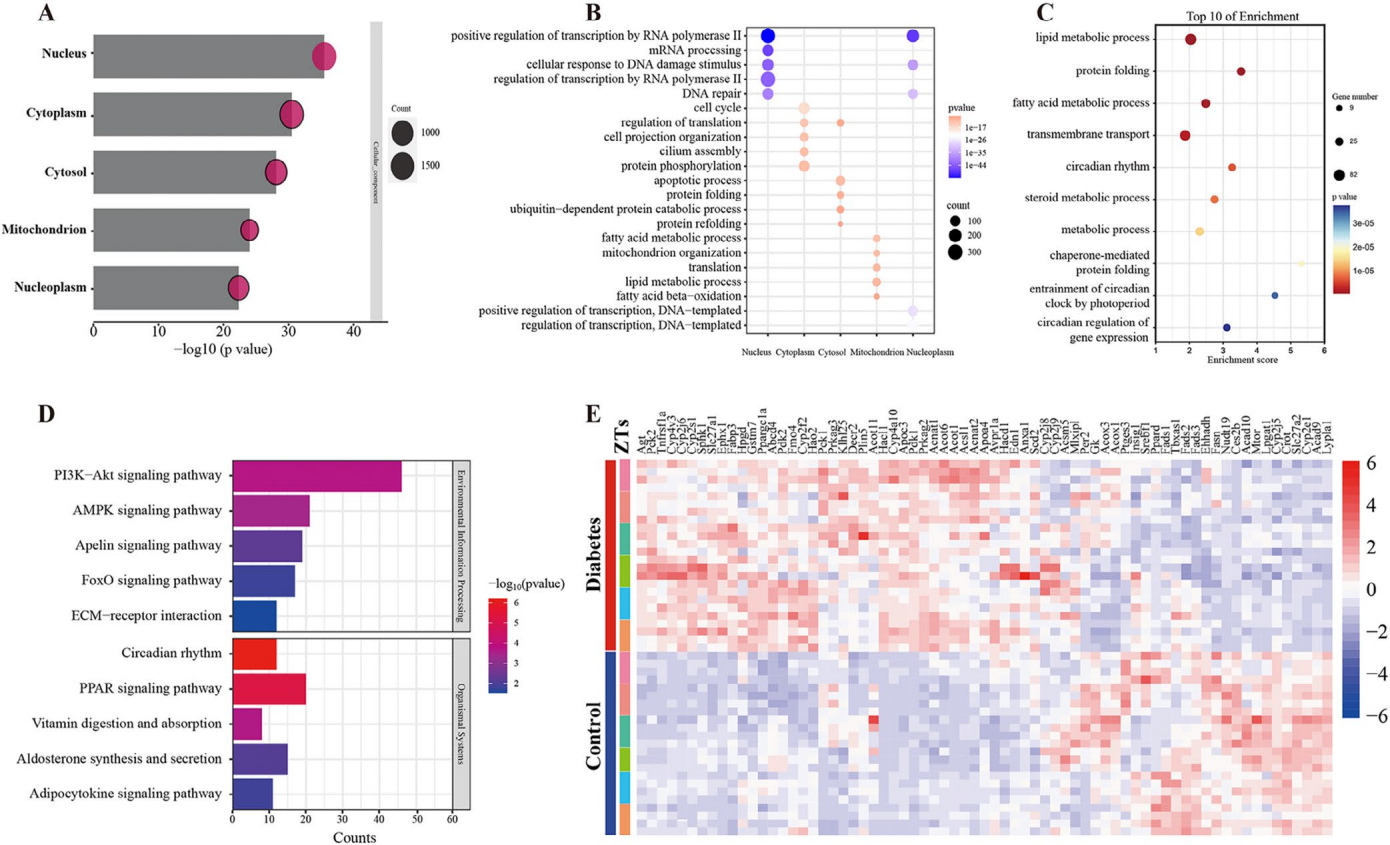
Circadian dysregulation of lipid metabolism is a prominent feature in the pathogenesis of diabetic nephropathy. The bar plot displays the top 5 cellular components by GO enrichment analysis performed on the circadian‐dysregulation gene set. (B) The categorized GO bubble plot shows the top 5 enriched biological processes in the corresponding cellular components, respectively. (C) GO bubble plot illustrates the top 10 biological processes enriched among genes exhibiting differential expression of at least one ZT in the circadian dysregulation gene set. (D) The KEGG bar plot demonstrates the top 10 KEGG pathways enriched among genes exhibiting differential expression at least at one ZT in the circadian dysregulation gene set. (E) Heatmap exhibits the fatty acid metabolic process related genes. ZT, zeitgeber time.

The group of genes that either lose, acquire, or maintain rhythmicity in diabetic mice is collectively referred to as the circadian dysregulation gene set. GO enrichment analysis was performed on this gene set and the top 5 cellular components are illustrated in Figure 5A. The result indicates that the rhythmic disturbance primarily occurs at the nucleus, cytoplasm, cytosol, mitochondrion, and nucleoplasm in diabetic nephropathy. Specifically, the most significant circadian perturbations in the corresponding cellular components primarily involve positive regulation of transcription by RNA polymerase II, cell cycle, regulation of translation, fatty acid metabolic process, and positive regulation of transcription by RNA polymerase II, respectively (Figure 5B). To further identify individuals with dysregulated circadian rhythms that may play a critical role in the pathogenesis of diabetic nephropathy, we next screened for genes within the circadian dysregulation gene set that exhibit differential expression at least at one ZT. A total of 1490 genes were identified (Table S2). In terms of GO enrichment analysis, they were mainly enriched in lipid metabolic process, protein folding, fatty acid metabolic process, transmembrane transport, and circadian rhythm (Figure 5C). As for KEGG analysis, significant enrichment was observed in pivotal pathways such as the AMPK signaling pathway and the PPAR signaling pathway (Figure 5D), crucial for fatty acid metabolism regulation. Seventy genes associated with the fatty acid metabolic process were extracted from the pool of 1490 genes using the MSigDB (MM4790) (Figure 5E). These results suggest that circadian dysregulation of fatty acid metabolism might be a key pathogenic feature in diabetic nephropathy. Then our attention focused on investigating the circadian dysregulation of fatty acid metabolism.

### 3.6 The Implications of Circadian Dysregulation in Core Clock Genes and the PPAR Signaling Pathway on Fatty Acid Metabolism in Diabetic Nephropathy

**FIGURE 6:**
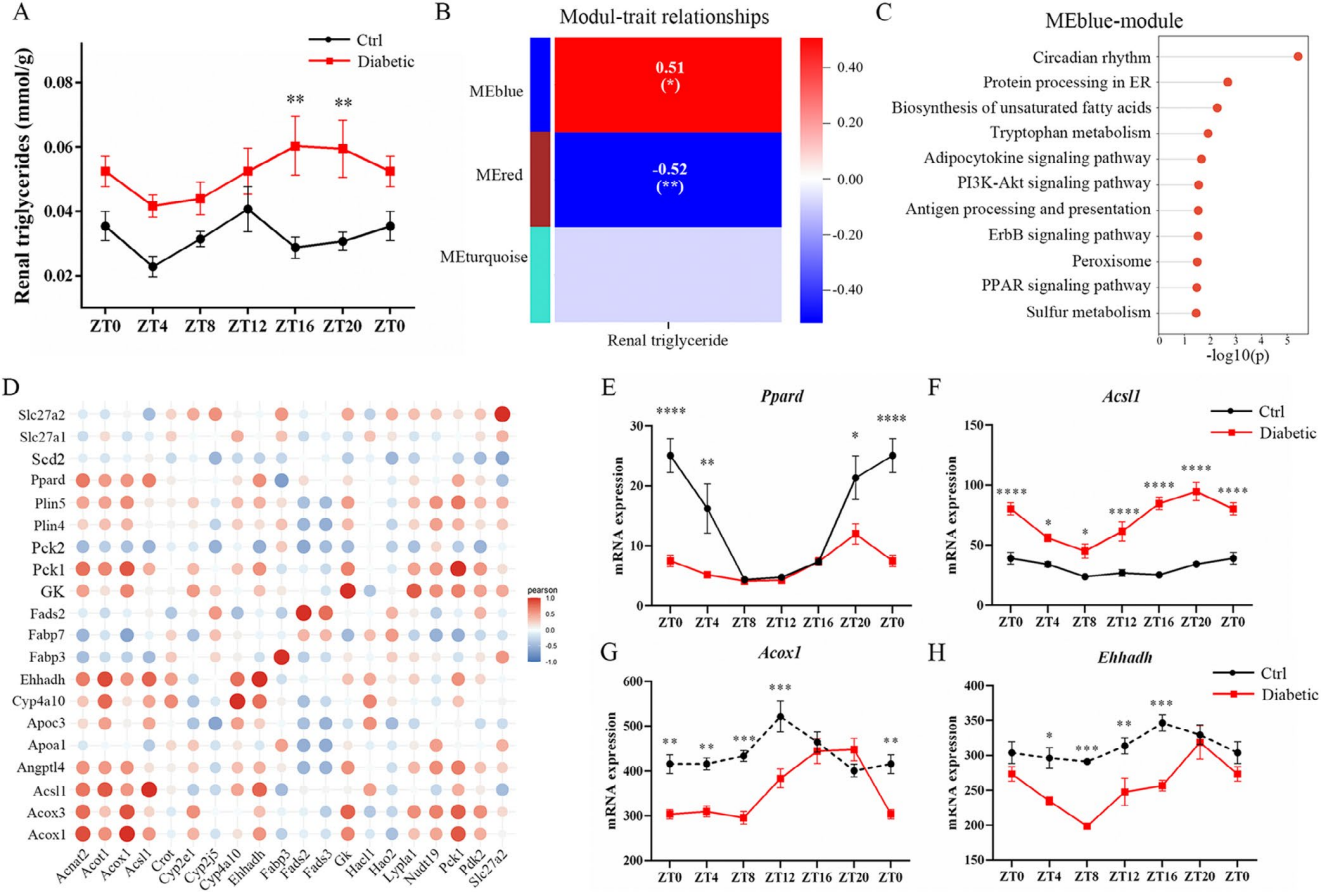
Correlation between core clock genes and PPAR signaling pathway with fatty acid metabolism in diabetic mice. (A) Time‐series levels of renal triglyceride in both control (black line) and diabetic mice (red line). Data are mean ± SEM. Two‐way ANOVA with Bonferroni multiple comparison correction was performed. **p < 0.01; (B) The WGCNA analysis reveals the relationship between module‐trait and renal triglyceride levels in diabetic mice within the circadian dysregulation gene set. Gray module was excluded as they did not belong to any identified co‐expression modules. Each square's top value indicates the correlation coefficient between the module and renal triglyceride, while the bottom value in parentheses represents the corresponding *p*‐value for this correlation. (C) The horizontal lollipop chart displays the KEGG pathways enriched in the MEblue‐module. (D) The correlation matrix plot illustrates the relationship between the PPAR signaling pathway and fatty acid metabolism. Circadian expressions of (E) *Ppard*, (F) *Acsl1*, (G) *Acox1* and (H) *Ehhadh* in control (black line) and diabetic (red line) mice during 24 h. The solid line denotes rhythmic oscillation, while the dotted line represents arrhythmic. Data are mean ± SEM. Two‐way ANOVA with Bonferroni multiple comparison correction was performed and *p* value < 0.05 was considered significant. *p < 0.05; **p < 0.01; ***p < 0.001; ****p < 0.0001.

We conducted a detailed analysis to explore the potential regulatory relationship of fatty acid metabolism in diabetic nephropathy from the perspective of circadian. Time‐series levels of renal triglyceride were measured in both control and diabetic mice. In the control mice, the renal triglyceride levels exhibited oscillations throughout the day, peaking around ZT12. Compared with control mice, the diabetic mice showed elevated levels of renal triglycerides with distinct diurnal variations, with a peak at ZT16 (Figure 6A). Then, WGCNA was conducted on the circadian dysregulation gene set, aiming to identify pathways co‐expressed with renal triglycerides in different ZTs (Figure 6B). The analysis revealed that the MEblue‐module was enriched for genes involved in circadian rhythm and the PPAR signaling pathway (Figure 6C), suggesting the circadian dysregulation of renal triglyceride is functionally linked to the core clock genes and the PPAR signaling pathway in diabetic nephropathy. Subsequent co‐expression analysis further confirmed a correlation between the circadian expression of the genes linked to fatty acid metabolism and those associated with PPAR signaling pathways (Figure 6D). Additionally, several key genes involved in the PPAR signaling pathway and fatty acid beta‐oxidation, including *Ppard*, *Acsl1*, *Acox1*, and *Ehhadh*, showed similar circadian patterns in diabetic nephropathy (Figure 6E–H). These findings highlight the potential link between the circadian perturbation of core clock genes and the PPAR signaling pathway on fatty acid metabolism in diabetic nephropathy.

## 4 Discussion

Through analyzing the 24‐h oscillation of the renal transcriptome in both control and diabetic mice, our study revealed several aspects of the role of circadian rhythms in diabetic nephropathy. Firstly, we detected substantial circadian disruption in the kidneys of diabetic mice, characterized by both the gain and loss of rhythmicity, along with alterations in the phase and relative amplitude of genes that retained oscillatory expression. To elucidate the underlying contributors to these distinct circadian patterns in diabetic nephropathy, the circadian expression patterns of renal clock genes were examined. A phase advance in the expression of *Bmal1*, *Per3*, *Nr1d1*, *Nr1d2*, and *Dec1* in diabetic mice was observed. Additionally, key circadian rhythm parameters such as amplitude and mesor of several clock genes were altered, which may lead to changes in the circadian pattern of downstream clock‐controlled genes. Since the circadian rhythm of renal core clock genes was not drastically modified, extra‐renal signals may also contribute to the renal circadian disarrangement observed in diabetic nephropathy. One possible trigger is the feeding pattern change in diabetic mice. In control mice, the food was primarily consumed during the nocturnal phase, while db/db mice ingested food throughout the day, resulting in a higher amount of food intake and the significantly dampened feeding diurnal rhythm [18]. Numerous studies have revealed that feeding is an important synchronizer of metabolic processes in peripheral clocks [19]. For instance, a high‐fat diet in mice disrupted the rhythmic expression of clock‐controlled genes that regulate fuel metabolism in the hypothalamus, liver, and adipose tissue [20]. Besides, other potential triggers like the sleep–wake behavior alteration in db/db mice [21] may also participate in the dampened circadian rhythm in diabetic nephropathy.

Given the remarkable changes in the circadian output of diabetic mice, further analyses were conducted to elucidate the underlying influences. We discovered that in control mice, protein homeostasis and glycolipid metabolism were tightly coupled to circadian cues. However, this temporally integrated manner was disrupted in diabetic nephropathy. The advantages of circadian gene expression in physiology and disease continue to be a compelling area of exploration, with energy conservation being a key theoretical perspective [22]. Wang et al. [23] demonstrated that circadian gene expression serves as a strategic mechanism to optimize and minimize overall cellular energy expenditure. Therefore, circadian disruption of protein homeostasis and glycolipid metabolism may lead to increased energy expenditure and heightened mitochondrial stress in diabetic nephropathy. Additionally, investigators have confirmed that circadian regulation of certain physiological processes is critical for correct functioning. For instance, disrupting the circadian regulation of autophagy through the deletion of *Clock* in podocytes aggravated podocyte damage and enhanced proteinuria in diabetic nephropathy [15]. The regulation of *Clock* on mitochondrial autophagy is required for proper cardiomyocyte function in ischemic stress [24]. Furthermore, the circadian coupling of protein homeostasis and glycolipid metabolism may contribute to the maintenance of nutrient and energy homeostasis critical for normal function. For instance, we observed that the levels of autophagy‐related genes were highest during the late resting phase. This aligns with a previous study that demonstrated a similar diurnal rhythm of autophagy in high‐metabolic‐demand organs, including the liver, heart, muscle, and kidney [25]. Autophagy facilitates the degradation of damaged organelles to recycle essential nutrients, including amino acids, glucose, and fatty acids. Interestingly, the circadian peaks of genes related to gluconeogenesis and fatty acid β‐oxidation are closely coordinated with autophagic activity [26]. Remarkably, an elegant study by Song et al. [27] demonstrated that activating fatty acid β‐oxidation during the resting phase, which aligns with the substrate availability rhythm, improved heart failure in mice. In contrast, this effect was absent when fatty acid β‐oxidation was activated during the active phase. This finding underscores the critical role of temporal precision in metabolic disease interventions and highlights the significance of circadian regulation of autophagy in optimizing nutrient availability for storage or oxidative metabolism. Though additional research is needed to explore the underlying mechanism, our findings offer a novel circadian viewpoint on energy metabolism disorders in diabetic nephropathy.

To identify genes implicated in both circadian disturbance and transcriptional alterations in diabetic nephropathy, we screened for genes showing differential expression at least at one ZT in the circadian dysregulation gene set. Our analysis revealed that lipid metabolism, particularly fatty acid metabolism, is most prominent in the circadian disruption profile, indicating a potential impact of circadian dysregulation on lipid metabolism in the pathogenesis of diabetic nephropathy. In addition, using WGCNA and correlation analysis, the potential regulation of core clocks and the PPAR signaling pathway on fatty acid metabolism was revealed. Actually, the circadian control of lipid metabolism has been extensively studied. Numerous epidemiological investigations have documented a correlation between circadian misalignment (insufficient sleep duration and quality) and the escalating risk of obesity and diabetes [28, 29, 30, 31]. Studies utilizing the genetic mouse model have further confirmed the indispensable role of a proper circadian function for lipid metabolism. Global homozygous *Clock* mutant mice exhibited altered feeding rhythms, obesity, and other metabolic alterations such as hyperleptinemia, hyperlipidemia, hyperglycemia, and hypoinsulinemia [32]. Global *Bmal1*‐deficient mice displayed impaired glucose tolerance and increased lipid accumulation [33]. In addition, *Rev‐erbα* [34], *Per* [35], and *Cry* [36] are key regulators of lipid metabolism. Given that dyslipidemia is a well‐established contributor to diabetic nephropathy, causing renal damage both directly through lipotoxicity and indirectly via inflammation, oxidative stress, and mitochondrial dysfunction [37], the role of circadian regulation in lipid metabolism in diabetic nephropathy warrants greater attention and further exploration.

Given that our db/db mice model killed at 21 weeks old do not exhibit advanced lesions such as nodular sclerosis in the glomeruli, severe tubulointerstitial fibrosis, and tubular atrophy [38], our analyses should be interpreted solely as addressing the role of circadian rhythms in early‐stage diabetic nephropathy. As the disease progresses, the prevalence of cognitive impairment [39] and sleep disorder [40] increases, which may indicate a further dampening of circadian misalignment in diabetic nephropathy. Furthermore, since transcriptome analysis only detects mRNA oscillations, proteomic analysis and post‐translational modifications, such as the phosphorylome, are essential for studying the role of circadian rhythms at the protein level in diabetic nephropathy. Besides, our analyses could only provide a preliminary view of the associations between various biological functions and circadian rhythms in diabetic nephropathy. Direct evidence that circadian modulation of these processes influences disease pathogenesis remains to be established. Finally, whether the circadian observations derived from the diabetic nephropathy mouse model can be extrapolated to human diabetic nephropathy needs further validation.

Overall, we demonstrate that in normal conditions, a large proportion of renal genes exhibit rhythmic expression, a pattern that is significantly disrupted in diabetic nephropathy. The alterations in extra‐renal circadian signals and renal core clock genes likely have a combined effect on circadian disruption in diabetic nephropathy. Besides, we found the circadian pattern of a variety of biological processes, especially protein homeostasis and glycolipid metabolism, was disrupted in diabetic nephropathy.

Circadian dysregulation of the PPAR signaling pathway and lipid metabolism might play a critical role in the pathogenesis of diabetic nephropathy. Overall, our study reveals a significant link between circadian rhythms and diabetic nephropathy and further provides a new perspective for the intervention of dyslipidemia induced by circadian disorder in diabetic nephropathy.

## Author Contributions

Conceptualization, Zhan‐Zheng Zhao, Xiao‐Qian Li; Funding acquisition, Zhan‐Zheng Zhao, Xiao‐Qian Li; Animal housing and tissue collection, Xiao‐Qian Li, Yi‐Nuo Ma, Lu‐Yao Wang, Xiao‐Hui Li; Data analysis, Xiao‐Qian Li, Ting‐Yu Fu; Experiments, Xiao‐Qian Li, Tian‐Fen Chen; Writing – original draft, Xiao‐Qian Li; Writing – review and editing, Zhan‐Zheng Zhao, Jing Xiao, Xiao‐Qian Li, Lei Cheng.

## Funding

This study was supported by the Medical Science and Technology Joint Project of Henan Province (LHGJ20220296); the Scientific Research and Innovation Team of The First Affiliated Hospital of Zhengzhou University [grant number ZYCXTD2023009]; the Postdoctoral Research Initiation Fund. The National Natural Science Foundation of China (Grant No. 82500907). The funders had no role in study design, data collection and analysis, decision to publish, or preparation of the manuscript.

## Ethics Statement

This study was approved by the Animal Ethics Committee of the First Affiliated Hospital of Zhengzhou University (approval No. ZZU‐LAC20210604[09]).

## Consent

The authors have nothing to report.

## Conflicts of Interest

The authors declare no conflicts of interest.

## Supporting information


**Figure S1:** fba270078‐sup‐0001‐FigureS1.tif.

**Table S1:** The Cosinor analysis of core clock genes.

**Table S2:** The genes within the circadian dysregulation gene set that exhibit differential expression at least at one zeitgeber time.

## Data Availability

Stored in repository.
