# Supplementary figures and images for: Circadian Reprogramming of Protein Homeostasis and Glycolipid Metabolism in Diabetic Nephropathy
Source: FASEB Bioadv. 2026 Feb 11;8(2):e70078. doi: 10.1096/fba.2025-00305 (PMC12892125; doi:10.1096/fba.2025-00305)

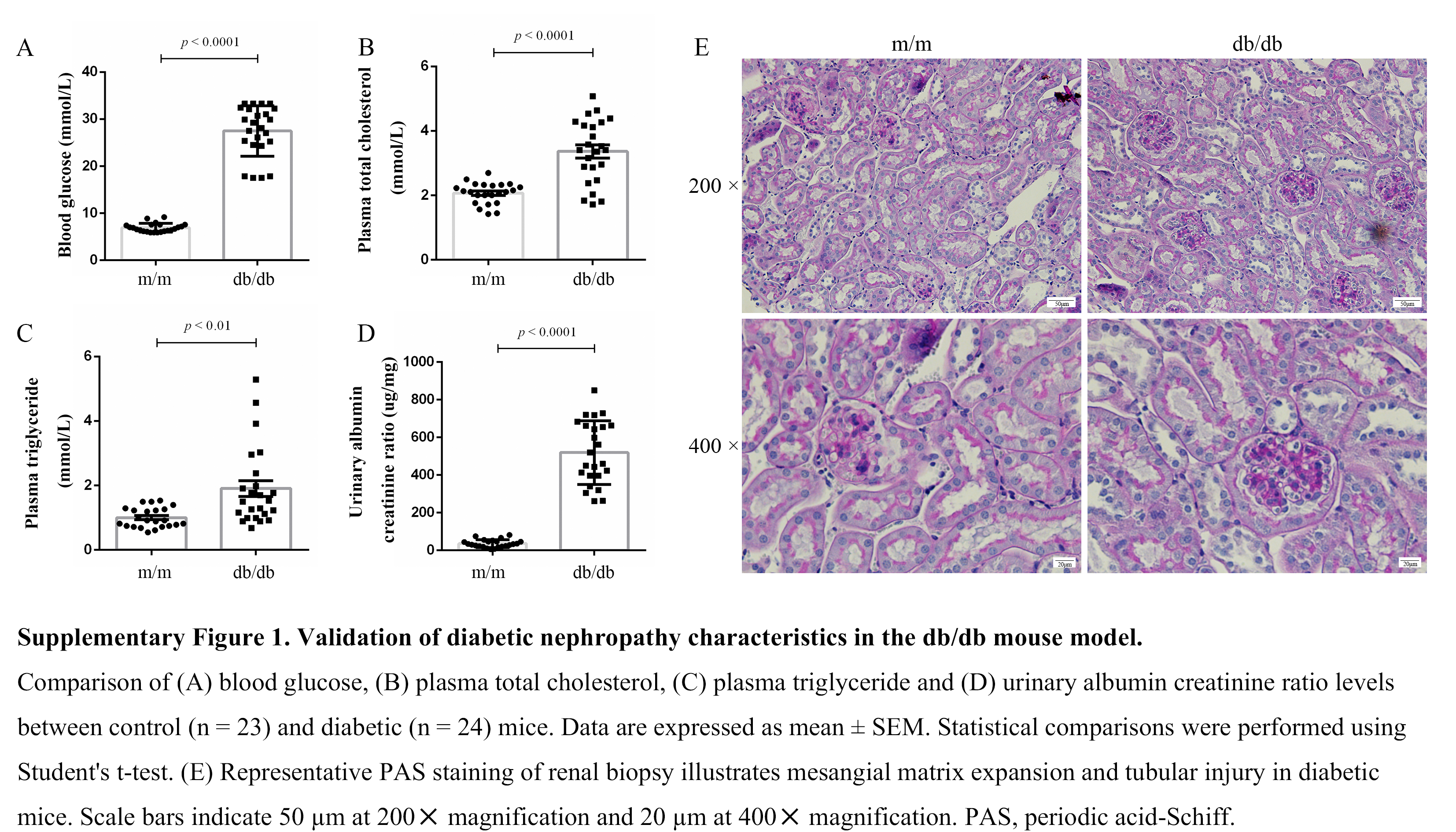

Supplement: Supplementary file 1 — Figure S1: fba270078‐sup‐0001‐FigureS1.tif. [file FBA2-8-e70078-s001.tif]
